# Single-Shot 3D Shape Reconstruction Using Structured Light and Deep Convolutional Neural Networks

**DOI:** 10.3390/s20133718

**Published:** 2020-07-03

**Authors:** Hieu Nguyen, Yuzeng Wang, Zhaoyang Wang

**Affiliations:** 1 Department of Mechanical Engineering, The Catholic University of America, Washington, DC 20064, USA; 2 Neuroimaging Research Branch, National Institute on Drug Abuse, National Institutes of Health, Baltimore, MD 21224, USA; hieu.nguyen@nih.gov; 3 School of Mechanical Engineering, Jinan University, Jinan 250022, China; me_wangyz@ujn.edu.cn

**Keywords:** three-dimensional image acquisition, three-dimensional sensing, three-dimensional shape reconstruction, depth measurement, structured light, fringe projection, convolutional neural networks, deep machine learning

## Abstract

Single-shot 3D imaging and shape reconstruction has seen a surge of interest due to the ever-increasing evolution in sensing technologies. In this paper, a robust single-shot 3D shape reconstruction technique integrating the structured light technique with the deep convolutional neural networks (CNNs) is proposed. The input of the technique is a single fringe-pattern image, and the output is the corresponding depth map for 3D shape reconstruction. The essential training and validation datasets with high-quality 3D ground-truth labels are prepared by using a multi-frequency fringe projection profilometry technique. Unlike the conventional 3D shape reconstruction methods which involve complex algorithms and intensive computation to determine phase distributions or pixel disparities as well as depth map, the proposed approach uses an end-to-end network architecture to directly carry out the transformation of a 2D image to its corresponding 3D depth map without extra processing. In the approach, three CNN-based models are adopted for comparison. Furthermore, an accurate structured-light-based 3D imaging dataset used in this paper is made publicly available. Experiments have been conducted to demonstrate the validity and robustness of the proposed technique. It is capable of satisfying various 3D shape reconstruction demands in scientific research and engineering applications.

## 1. Introduction

Non-contact 3D shape reconstruction using the structured-light techniques is commonly used in a broad range of applications including machine vision, reverse engineering, quality assurance, 3D printing, entertainment, etc. [1,2,3,4,5,6,7,8,9,10,11]. The technique typically retrieves the depth or height information with an algorithm based on geometric triangulation, where the structured light helps facilitate the required image matching or decoding process. According to the number of images required for each 3D reconstruction, the structured-light techniques can be classified into two categories: multi-shot [12,13,14,15] and single-shot [16,17,18]. The multi-shot techniques are capable of capturing high-resolution 3D images at a limited speed and are thus widely used as industrial metrology for accurate shape reconstructions. By contrast, the single-shot techniques can acquire 3D images at a fast speed to deal with dynamic scenes and are receiving tremendous attention in the fields of entertainment and robotics. As technologies evolve at an ever-increasing pace, applying the concept of deep machine learning to the highly demanded single-shot 3D shape reconstructions has now become feasible.

In the machine learning field, deep convolutional neural networks (CNNs) have found numerous applications in object detection, image classification, scene understanding, medical image analysis, and natural language processing, etc. The recent advances of using the deep CNNs for image segmentation intend to make the network architecture an end-to-end learning process. For example, Long et al. [19] restored the downsampled feature map to the original size of the input using backwards convolution. An impressive network architecture, named UNet and proposed by Ronneberger et al. [20], extended the decoding path from Long’s framework to yield a precise output with a relatively small number of training images. Similarly, Badrinarayanan et al. [21] used an idea of upsampling the lowest of the encoder output to improve the resolution of the output with less computational resources.

In the CNN-based 3D reconstruction and depth detection applications, Eigen et al. [22] and Liu et al. [23] respectively proposed a scheme to conduct the depth estimation from a single view using the CNNs. In their work, they used a third-party training dataset produced by Kinect RGB-D sensors, which has low accuracy and is insufficient for good learning. Inspired by these two methods, Choy et al. [24] proposed a novel architecture which employs recurrent neural networks (RNNs) among the autoencoder CNNs for single- and multi-view 3D reconstructions. Later, Duo et al. [25] proposed a deep CNN scheme to reconstruct the 3D face shape from a single facial image. Although Duo’s framework showed outstanding performance with a synthetic and publicly available database, it requires a post-processing step which further relies on combining a set of shape and blend-shape basis. Recently, Paschalidou et al. [26] proposed an end-to-end Raynet to reconstruct dense 3D models from multiple images by combining the CNNs with a Markov random field. A common issue with these existing techniques is the lack of using high-accuracy training data.

Over the past year, the utilizing of the CNN frameworks for fringe analysis has become active in the optics field. For instance, Feng et al. [27,28] integrated the deep CNNs with the phase-shifting scheme for unwrapped phase detections. Yin et al. [29] removed the phase ambiguities in the phase unwrapping process with two groups of phase-shifted fringe patterns and deep learning. Jeught [30] proposed a neural network with a large simulated training dataset to acquire the height information from a single fringe pattern. A number of other investigations [31,32,33,34,35] have also shown promising results on using the CNN models to improve the estimation and determination of phase distributions. In addition, various techniques have been proposed to reduce the noise in fringe pattern analysis using deep learning schemes [36,37,38].

**Figure 1:**
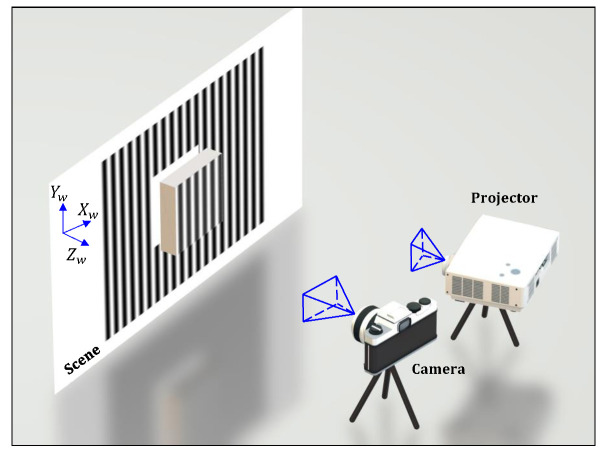
Schematic of the FPP-based 3D imaging and shape measurement system.

Based on the successful applications of the CNNs to image segmentation, 3D scene reconstruction, and fringe pattern analysis, the exploration of utilizing the deep CNNs to accurately reconstruct the 3D shapes from a single structured-light image should be quite viable. With numerous parameters, a deep CNN model can be trained to approximate a very complex non-linear regressor that is capable of mapping a conventional structured-light image to its corresponding 3D depth or height map. At present, the robustness and importance of integrating the deep CNNs with one of the most widely used structured-light methods, fringe projection profilometry (FPP) technique, have started drawing lots of attention and are emerging quickly. In this paper, such integration for accurate 3D shape reconstruction is investigated in a different perspective. The main idea is to transform a single-shot image, which has a high-frequency fringe pattern projected on the target, into a 3D image using a deep CNN that has a contracting encoder path and an expansive decoder path. Compared with the conventional 3D shape measurement techniques, the proposed technique is considerably simpler without using any geometric information or any complicated registration and triangulation computation. A schematic of the single-shot FPP-based 3D system is shown in Figure 1.

Using real and accurate training data is essential for a reliable machine learning model. Because the FPP technique is one of the most accurate 3D shape measurement techniques and is able to perform 3D imaging with accuracy better than 0.1 mm, it is employed in this work to generate the required datasets for learning and evaluation. That is, the system shown in Figure 1 serves two purposes: it first provides the conventional FPP technique with multi-shot images (e.g., 16 images in each entry) to prepare the ground-truth data for the training and validation of the networks; then acquires single-shot images (i.e., one image in each entry) for the application of the proposed technique.

The rest of the paper is elaborated as follows. Section 2.1 reviews the FPP-based technique to generate high-accuracy training datasets. Section 2.2 describes the details of the proposed network architectures. Section 3 provides a few experimental results. Finally, Section 4 and Section 5 conclude the paper with discussions and a brief summary.

## 2. Methodology

### 2.1. Fringe Projection Profilometry (FPP) Technique for Training Data Generation

The most reliable FPP technique involves projecting a set of phase-shifted sinusoidal fringe patterns from a projector onto the objects, where the surface depth or height information is naturally encoded into the camera-captured fringe patterns for the subsequent 3D reconstruction process. Technically, the fringe patterns help establish the correspondences between the captured image and the original reference image projected by the projector. In practice, the technique reconstructs the 3D shapes through determining the height or depth map from the phase distributions of the captured fringe patterns. The phase extraction process normally uses phase-shifted fringe patterns to calculate the fringe phase. In general, the original fringes are straight, evenly spaced, and vertically (or horizontally) oriented. They are numerically generated with the following function [39,40,41,42]:

(1) $$I_{j}^{\left(p\right)}\left(u,v\right)=I_{0}\left[1+cos(\phi +\delta_{j})\right]=I_{0}^{\left(p\right)}\left[1+cos(2\pi f\frac{u}{W}+\delta_{j})\right]$$

where $I^{\left(p\right)}$ is the intensity value of the pattern at pixel coordinate (u,v); the subscript *j* denotes the *j*th phase-shifted image with $j=\left\{1,2,\ldots ,m\right\}$, and m is the number of the phase-shift steps (e.g., m=4); $I_{0}^{\left(p\right)}$ is a constant coefficient indicating the value of intensity modulation; *f* is the number of fringes in the pattern; *W* is the width of the generated image; δ is the phase-shift amount; and ϕ is the fringe phase.

The projector projects the fringe patterns onto the target of interest, and the camera captures the images of the target with projected fringes. The phase distribution in the images can be calculated by using a standard phase-shifting algorithm, typically the four-step one. The phase ϕ at a pixel (u,v) can be determined as:

(2) $$\phi^{w}\left(u,v\right)=\arctan\frac{I_{4}\left(u,v\right)-I_{2}\left(u,v\right)}{I_{1}\left(u,v\right)-I_{3}\left(u,v\right)},$$

In the equation, I(u,v) indicates the intensity value at the pixel coordinate (u,v) in the captured images, and the subscript numbers 1–4 represents the sequential steps of the four phase-shifted patterns in the images.

It can be seen from the equation that the phase value is wrapped in a range of 0 to 2π (denoted with a superscript *w*) and must be unwrapped to obtain the true phase. In order to cope with the phase-unwrapping difficulty encountered in the cases of complex shapes and geometric discontinuities, a scheme of using multi-frequency fringe patterns is often employed in practice. The corresponding unwrapped phase distributions can be calculated from [43,44,45]:

(3) $$\phi_{i}\left(u,v\right)=\phi_{i}^{w}\left(u,v\right)+\mathrm{INT}\left(\frac{\phi_{i-1}\frac{f_{i}}{f_{i-1}}-\phi_{i}^{w}}{2\pi }\right)2\pi$$

where ϕ is the unwrapped phase; *i* indicates the *i*th fringe-frequency pattern with $i=\left\{2,3,\ldots ,n\right\}$, and *n* is the number of fringe frequencies; INT represents the function of rounding to the nearest integer; $f_{i}$ is the number of fringes in the *i*th projection pattern, with $f_{n}>f_{n-1}>\ldots >f_{1}=1$; and $\phi_{1}=\phi_{1}^{w}$ is satisfied for $f_{1}=1$. The ratio between two adjacent fringe frequencies $\frac{f_{i}}{f_{i-1}}$ is normally smaller or equal to 5 to reduce the noise effect and ensure the reliability of the algorithm. A practical example is n=4 with $f_{4}=100$, $f_{3}=20$, $f_{2}=4$, and $f_{1}=1$. It can be seen that four images for each frequency and four frequencies for each measurement indicate a total of 16 images for each accurate FPP measurement.

The essential task of the FPP technique is to retrieve the depth or height map from the calculated phase distributions of the highest frequency fringes with the highest possible accuracy. The governing equation for a generalized setup where the system components can be arbitrarily positioned [46,47] is:

(4) $$\begin{array}{cc} z_{w} & =\frac{Cp^{⊺}}{Dp^{⊺}}\\ C & =\left\{1\;\;c_{1}\;\;c_{2}\;\;c_{3}\;\;\cdots \;\;c_{17}\;\;c_{18}\;\;c_{19}\right\}\\ D & =\left\{d_{0}\;\;d_{1}\;\;d_{2}\;\;d_{3}\;\;\cdots \;\;c_{17}\;\;d_{18}\;\;d_{19}\right\}\\ p & =\left\{1\;\;\phi \;\;u\;\;u\phi \;\;v\;\;v\phi \;\;u^{2}\;\;u^{2}\phi \;\;uv\;\;uv\phi \;\;v^{2}\;\;v^{2}\phi \;\;u^{3}\;\;u^{3}\phi \;\;u^{2}v\;\;u^{2}v\phi \;\;uv^{2}\;\;uv^{2}\phi \;\;v^{3}\;\;v^{3}\phi \right\} \end{array}$$

where $z_{w}$ is the height or depth at the point corresponding to the pixel (u,v) in the captured images, and it is also the z-coordinate of the point in the reference or world coordinate system; ϕ is the unwrapped phase of the highest-frequency fringe pattern at the same pixel; and $c_{1}–c_{19}$ and $d_{0}–d_{19}$ are constant coefficients associated with geometrical and other system parameters. The 39 coefficients can be determined by a calibration process using a few gage objects that have many points with $z_{w}$ precisely known. After the height or depth map is obtained using Equation (4), the other two coordinates $x_{w}$ and $y_{w}$ can be easily determined upon knowing the camera and lens parameters. For this reason, the two terms, depth measurement and 3D shape reconstruction, can be often used interchangeably.

The FPP technique is employed to generate the training datasets, including the validation data. Once the training datasets are available, they can be directly fed into deep CNN models for subsequent learning process. It is noteworthy that the FPP technique is also used to provides the ground-truth results of the test dataset for evaluation purpose.

### 2.2. Network Architecture

**Figure 2:**
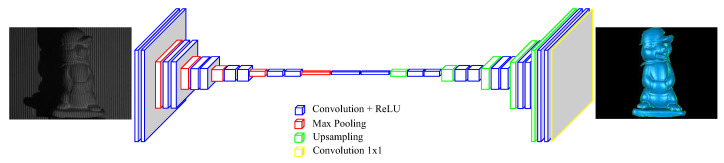
Illustration of the single-shot 3D shape reconstruction system using FPP and CNNs.

Given a single-shot input image of an object or a few objects, the proposed approach uses a deep neural network to transform the image into a 3D point cloud from the fringe patterns presented in the image. Figure 2 illustrates how the proposed integration of fringe projection with deep machine learning works. The adopted network is mainly made up of two components: the encoder path and the decoder path. The encoder path includes convolution and pooling operations that are capable of detecting essential features from the input image. The decoder path, on the other hand, contains transpose convolution and unpooling operations that can stack and concatenate lower resolution feature maps to form higher resolution layers. The output of the network is a 3D depth or height map corresponding to the input image.

Three different deep CNNs are adopted in this work for comparison to find the best approach. The three networks are as follows:

- **Fully convolutional networks (FCN).** The FCN is a well-known network for semantic segmentation. FCN adopts the encoder path from the contemporary classification networks and transforms the fully connected layers into convolution layers before upsampling the coarse output map to the same size as the input. The FCN-8s architecture [19] is adopted in this paper to prevent the loss of spatial information, and the network has been modified to work with the input image and yield the desired output information of depth.
- **Autoencoder networks (AEN).** The AEN has an encoder path and a symmetric decoder path. The proposed AEN has totally 33 layers, including 22 standard convolution layers, 5 max pooling layers, 5 transpose operation layers, and a 1×1 convolution layer.
- **UNet.** The UNet is also a well-known network [20], and it has a similar architecture to the AEN. The key difference is that in the UNet the local context information from the encoder path is concatenated with the upsampled output, which can help increase the resolution of the final output.

**Figure 3:**
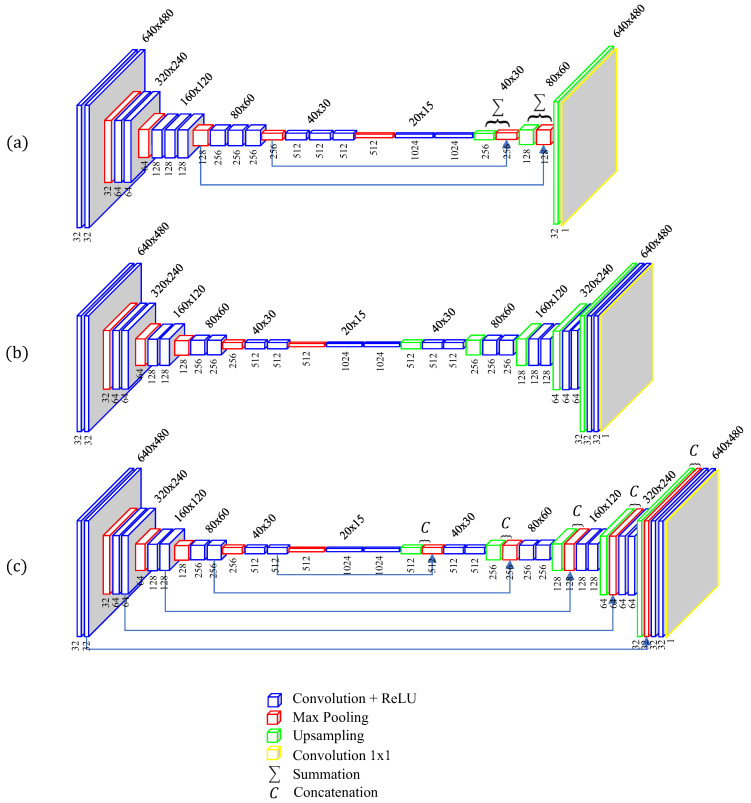
The proposed network architectures: (**a**) Fully convolutional networks (FCN), (**b**) Autoencoder networks (AEN), and (**c**) UNet.

The architectures of the three deep CNNs are shown in Figure 3.

In the learning process of the three CNNs, the training or validation dataset in each CNN model is a four-dimensional array of size s×h×w×c, where *s* is the number of the data samples; *h* and *w* are the spatial dimensions or image dimensions; *c* is the channel dimension, with c=1 for grayscale images and c=3 for color images. The networks contain convolution, pooling, transpose convolution, and unpooling layers; they do not contain any fully connected layers. Each convolution layer learns the local features from the input and produces the output features where the spatial axes of the output map remain the same but the depth axis changes following the convolution operation filters. A nonlinear activation function named rectified linear unit (ReLU), expressed as max(0,x), is employed in each convolution layer.

In the chain-based architecture, each layer in the network is given by [48]

(5) $$h=g(W^{⊺}x+b)$$

where h is the output vector; function *g* is called an activation function; x is a vector of input; the parameters W in a matrix form and b in a vector form are optimized by the learning process.

The max pooling layers with a 2×2 window and a stride of 2 are applied to downsample the feature maps through extracting only the max value in each window. In the AEN and UNet, the 2D transpose convolution layers are applied in the decoder path to transform the lower feature input back to a higher resolution. Finally, a 1×1 convolution layer is attached to the final layer to transform the feature maps to the desired depth or height map. Unlike the conventional 3D shape reconstruction schemes that often require complex algorithms based on a profound understanding of techniques, the proposed approach depends on numerous parameters in the networks, which are automatically trained, to play a vital role in the single-shot 3D reconstruction.

## 3. Experiments and Results

An experiment has been conducted to validate the proposed approach. The experiment uses a desktop computer with an Intel Core i7-980 processor, a 16-GB RAM, and a Nvidia GeForce GTX 1070 graphics card as well as an Epson PowerLite98 projector and a Silicon Video 643M camera. Keras, a popular Python deep learning library, is utilized in programming implementation. In addition, Nvidia’s cuDNN deep neural network library is adopted to speed up the training process. The field of view of the experiment is about 155 mm, and the distance from the camera to the scene of interest is around 1.2 m. This working distance is typical in real applications of the 3D shape measurement, and it can be substantially shorter or longer without affecting the nature of the proposed work. In the experiments, a number of small plaster sculptures serve as the objects, whose sizes and surface natures are suitable for producing reliable and accurate 3D datasets.

### 3.1. Training and Test Data Acquisition

Unlike the synthetic data adopted by plenty of recent research, three different types of CNNs have been tested using real collected training data. The experiment uses four fringe frequencies (1, 4, 20, and 100) and the four-step phase-shifting schemes, which usually yield a good balance among accuracy, reliability, and capability. The first image of the last frequency (i.e., $f_{4}=100$) is chosen as the input image, and all other 15 images are captured solely for the purpose of creating the ground-truth 3D height labels. Totally, the experiment generated 1120, 140, and 140 samples in the training, validation, and test datasets, respectively; and each sample contains a single fringe image of the object(s) and a corresponding height map. The data split ratio is 80%–10%–10% and is appropriate for such a case of small datasets. The datasets have been made publicly available for download (Ref. [49]). Supplementary Video S1 shows the input images and the ground-truth labels of the training and test data side by side. It is noted that the background is neither mandatory nor has to be flat, and it is shown in the input images but hidden in the visualization for better demonstration purposes. Moreover, the original shadow areas are excluded from the learning process.

### 3.2. Training, Analysis, and Evaluation

The training and validation data are applied to the learning process of the FCN, AEN, and UNet models. The optimization Adam [50] adopts a total of 300 epochs with a mini-batch size of 2 images. The learning rate is reduced by half whenever the validation loss does not improve within 20 consecutive epochs. A few regularization schemes, including data augmentation, weight regularization, data shuffling, and dropout, are employed to tackle the over-fitting problem. Furthermore, the grid search method is conducted to obtain the best hyperparameters for the training model. In order to check the network performance as the training process iterates, a callback is performed at the end of each epoch on a randomly pre-selected test image to predict the 3D shapes using the updated model parameters (see Supplementary Video S2). The learning can adopt either the binary cross-entropy or the mean squared error as the loss function. If the loss function is set as binary cross-entropy, the ground truth data is scaled back to the value range between 0 and 1.

The evaluation is carried out by calculating the mean relative error (MRE) and the root mean squared error (RMSE) of the reconstructed 3D shapes. Table 1 shows the performance errors of the three CNN models for single-shot 3D shape reconstruction. It can be seen that the FCN model yields the largest error among the three CNNs, and its learning time is also the longest because of the involved element-wise summation. The AEN model requires the least learning time, but its performance is slightly inferior to that of the UNet in terms of accuracy. It is noticed that the network generally performs better when the models are trained through a smaller batch size.

**Figure 4:**
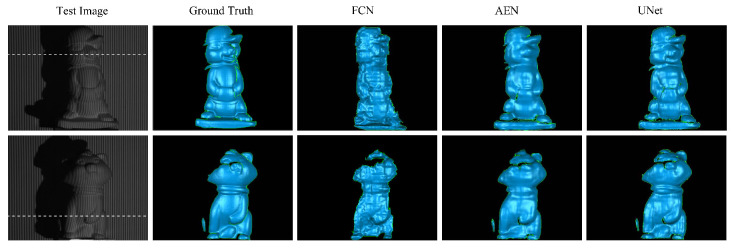
3D reconstruction results of two representative test images.

**Figure 5:**
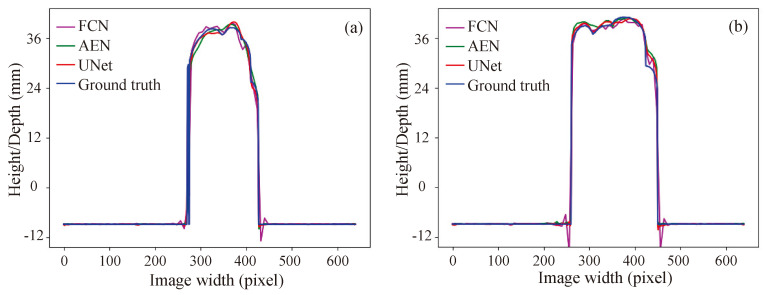
Height distributions: (**a**) along a line highlighted in the first test image in Figure 4, (**b**) along a line highlighted in the second test image in Figure 4.

Figure 4 demonstrates a visual comparison of the ground-truth 3D data and the reconstructed 3D data acquired with the three networks. The first image in each row is a representative input, and the next is the corresponding 3D ground-truth image. The following three images in each row are the reconstructed results from the FCN, AEN, and UNet models, respectively. Figure 5 shows the height distributions relative to the background plane along an arbitrary line highlighted in each of the initial input image. Again, it is evident from Figure 4 and Figure 5 that the AEN and UNet models perform better than the FCN model. The main reason is that the FCN abruptly restores the high-resolution feature map from the lower one by using the bilinear upsampling operators, consequently many details are lacking in the final reconstructed 3D results. The AEN and UNet each consists of its decoder path that is symmetric to the encoder path, which helps steadily propagate the context information between layers to produce features depicting detailed information. Unlike the AEN, the UNet contains the concatenation operation to send extra local features to the decoder path. This handling helps the network to perform the best among the three networks.

**Figure 6:**
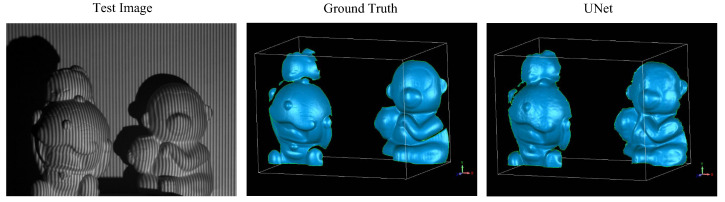
3D reconstruction results of multiple separated objects.

Reconstructing the 3D shapes of multiple separated objects at different depths is a task that cannot be fulfilled by typical single-shot fringe projection methods because of the fringe phase discontinuity issue. Nevertheless, the surface discontinuity is not a problem for the proposed approaches since the CNN models learn from datasets with labels directly and do not involve phase calculation. An example of the 3D shape reconstruction of multiple separate objects using the UNet model is shown in Figure 6.

**Figure 7:**
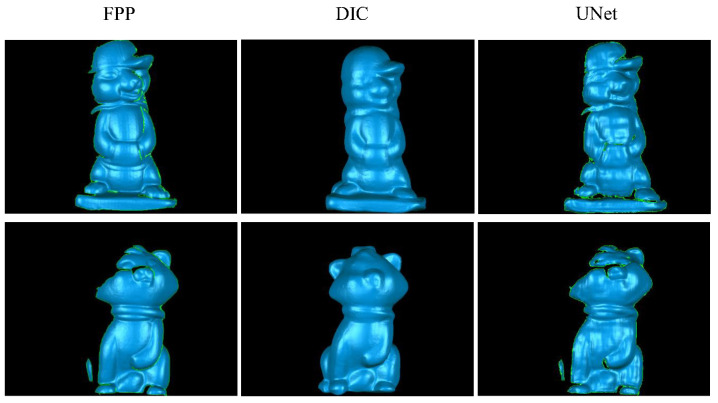
Examples of the 3D shape reconstruction results acquired by using the FPP, the 3D-DIC, and the proposed UNet techniques.

A final experiment has been accomplished to demonstrate the performance comparison of the proposed UNet technique with two existing popular techniques: the conventional FPP technique and the 3D digital image correlation (3D-DIC) technique [51,52,53]. Technically, the 3D-DIC technique is considered as a single-shot stereo vision method as it requires simultaneously capturing two images from two different views with two cameras. The 3D-DIC technique relies on the area- or block-based image registration, so it generally yields measurement resolution lower than that provided by the FPP technique. Figure 7 displays the visual comparison of the 3D shape reconstructions obtained from three techniques. It is evident from the figure that the FPP technique gives the highest measurement accuracy, and the proposed UNet technique is slightly inferior to the 3D-DIC technique in terms of accuracy. It is noteworthy that the 3D reconstruction time for a new single image using the proposed method is generally less than 50 ms on the aforementioned computer, which indicates that a real-time 3D shape reconstruction is practicable. In contrast, the typical running times of the FPP and the 3D-DIC techniques are in the range of 0.1 to 2 s.

## 4. Discussions

In regard to the accuracy of the proposed techniques, although the performance could be further improved with much larger training datasets as well as deeper networks, preparing a considerably large number of high-accuracy ground truth data is very time-consuming and challenging at present. Furthermore, a deeper network will require a large amount of computer memory and computational time for the learning process. Similarly, the proposed techniques are technically suitable for high-resolution 3D shape reconstructions, but it is currently impractical because the learning time would be substantially longer and a much larger memory size would be mandatory. The future work can include exploring improved and advanced network models, preparing larger datasets with a robot-aided process, developing less memory-consuming algorithms, and using sophisticated central processing units (CPUs) and graphics processing units (GPUs) as well as cloud computing services such as Amazon Web Services, Google Cloud, Microsoft Azure Cloud, and IBM Cloud [54,55,56,57]. Machine learning just gains popularity in the recent a few years, and it is reasonable to believe that the aforementioned time-consuming and memory-consuming drawbacks will be lifted in the soon future.

The deep machine learning models use multi-layer artificial neural networks to approach the complex relationships between the captured images and physical quantities. Although the unprecedented behaviors cannot be rigorously explained at present, more details will be revealed as studies and investigations go further. The proposed techniques depend on using fringe-projection images, but it will be worthy exploring networks and algorithms capable of working with other kinds of structured-light images. Considering that the existing 3D imaging and shape measurement techniques normally require complicated algorithms and relatively long computation time, the novel 3D imaging and shape measurement approaches based on artificial networks and deep machine learning may have a considerable impact to the sensors and other relevant fields.

## 5. Conclusions

In summary, a novel single-shot 3D shape reconstruction technique is presented. The approach employs three deep CNN models, including FCN, AEN, and UNet, to quickly reconstruct the 3D shapes from a single image of the target with a fringe pattern projected on it. The learning process is accomplished through using the training and validation data acquired by a high-accuracy multi-shot FPP technique. Experiments show that the UNet performs the best among the three networks. The validity of the approach gives great promise in future research and development, which includes, but not limited to, using larger datasets and less memory-consuming algorithms as well as conducting a rigorous in-depth investigation on the CNN models.

The measurement accuracy of the proposed technique is currently inferior to the existing prevalent 3D imaging and 3D shape reconstruction techniques. Nevertheless, its unprecedented behavior, i.e., simple and robust, shows great potential in future high-performance 3D imaging. It could remarkably broaden the capabilities and applications of the 3D imaging and shape reconstruction in many fields of scientific research and engineering applications.

## Figures and Tables

**Table 1:** Performance evaluation of the CNNs.

Model		FCN	AEN	UNet
Training Time		7 h	5 h	6 h
Training	MRE	1.28 $\times \;10^{-3}$	8.10 $\times \;10^{-4}$	7.01 $\times \;10^{-4}$
	RMSE (mm)	1.47	0.80	0.71
Validation	MRE	1.78 $\times \;10^{-3}$	1.65 $\times \;10^{-3}$	1.47 $\times \;10^{-3}$
	RMSE (mm)	1.73	1.43	1.27
>Test	MRE	2.49 $\times \;10^{-3}$	2.32 $\times \;10^{-3}$	2.08 $\times \;10^{-3}$
	RMSE (mm)	2.03	1.85	1.62

## Supplementary Materials

The following two video clips are available online at https://www.mdpi.com/1424-8220/20/13/3718/s1, Video S1: Input images and their ground-truth labels of the training and test data, Video S2: Demonstration of the intermediate results of the training process after each epoch.
